# Impact of toothpaste on oral health-related quality of life in people with dentine hypersensitivity

**DOI:** 10.1186/s12903-019-0919-x

**Published:** 2019-10-22

**Authors:** Stephen Mason, Gary R. Burnett, Nisha Patel, Avinash Patil, Robert Maclure

**Affiliations:** 1 GSK Consumer Healthcare, St George’s Avenue, Weybridge, Surrey KT13 0DE UK; 2 Syneos Health, Commerzone, Building No. 4, 6th Floor, Survey No. 144/145, Samrat Ashok Path, Yerwada Jail Road, Pune, 411006 India; 3 Intertek CRS Ltd., 119 Station Road, Ellesmere Port, Cheshire, CH65 4BW UK

**Keywords:** Dentin sensitivity, Patient reported outcome measures, Pain, Tin fluoride, Dentin

## Abstract

**Background:**

Dentine hypersensitivity can impact functional status and everyday activities such as eating and talking. This study aimed to assess changes in oral health-related quality of life measures in individuals with dentine hypersensitivity following long-term use (24 weeks) of a commercially available toothpaste marketed for dentine hypersensitivity relief.

**Methods:**

This study was conducted across two sites and enrolled 75 adults with ≥2 non-adjacent sensitive teeth. Participants were assigned to twice-daily brushing with toothpaste containing 0.454% w/w stannous fluoride (1100 ppm fluoride). Every 4 weeks, participant-reported outcomes were assessed using the Dentine Hypersensitivity Experience Questionnaire (DHEQ), a condition specific oral health-related quality of life scale that has five domains and includes questions on social and emotional impact, restrictions, adaptations and effect on life overall. Responses to a clinically applied evaporative (air) stimulus were assessed using the examiner-observed Schiff sensitivity scale and Labelled Magnitude Scales (LMS), which included dentine hypersensitivity-specific descriptors of intensity, duration, tolerability and descriptive qualities of the participant’s response.

**Results:**

Participant-reported outcomes demonstrated reduction of the impact of dentine hypersensitivity over time on health-related quality of life, as measured by the DHEQ. This reached statistical significance from Week 8 onwards (*p* < 0.0001 versus baseline) for the Total DHEQ score, with scores continually decreasing at each timepoint. Most domain scores followed a similar pattern. Statistically significant reductions were also detected for the examiner-observed Schiff Sensitivity Scale scores at all timepoints (including at 4 weeks) (*p* < 0.05), which were mirrored by LMS responses. The toothpaste was generally well-tolerated.

**Conclusions:**

These results show that long-term use of a sensitivity toothpaste containing 0.454% w/w stannous fluoride has a beneficial, ongoing, impact on the oral health-related quality of life of people with dentine hypersensitivity.

**Trial registration:**

This study was registered at Clinicaltrials.gov (https://clinicaltrials.gov/ct2/show/NCT02752958) on April 27, 2016.

## Background

Dentine hypersensitivity (DH] is relatively common in adults, with a prevalence of between 12 and 42% [1, 2]. The defining symptom of DH is short, sharp pain unrelated to any other dental pathology or defect [3, 4]. This is typically assessed clinically by evaluating response to a potentially painful evaporative or tactile stimulus applied to the tooth, using either examiner-observed criteria (e.g., the Schiff Sensitivity Scale [5]) or participant-reported verbal descriptors and/or pain rating scales [6]. It is only recently that the wider psychosocial impacts of DH have been given much consideration. One qualitative study found that DH is experienced in complex ways in everyday life and has a wide variety of triggers and responses, not all of which are described as ‘pain’ [7]. Furthermore, DH impacts functional status and the ability to participate in everyday activities including eating, drinking, tooth brushing, talking and social interactions [7].

Oral health-related quality of life (OHrQoL) is a multidimensional construct [8]. Tools used to capture the impact of clinical interventions on OHrQoL are of increasing interest in dentistry [9]. Broad quality of life measures such as the Oral Health Impact Profile (OHIP) are commonly used [10, 11] and have shown that OHrQoL is significantly more impaired in those with DH than in the general population [12]. However, the generic OHIP is of limited value in assessing impact of DH interventions on OHrQoL as it doesn’t capture specific DH nuances [12]. The Dentine Hypersensitivity Experience Questionnaire (DHEQ) is a validated, condition-specific measure of OHrQoL in relation to DH [13, 14]. It was developed through a robust theoretical framework and has excellent internal and test–retest reliability [14]. The conception, development, validation and initial usage of the DHEQ has been published [15]. The measure has been validated with both long- and short-form versions, comprising 39 [14] and 15 [13] questions respectively, and has been translated into multiple languages (e.g., Chinese, Turkish, Portuguese) confirming its global relevance [16–19].

Another means of assessing DH from the affected individual’s perspective involves integration of verbal descriptors and numerical measurements to provide a richer description of the pain experience [20–22]. This approach has been refined to develop Labelled Magnitude Scales (LMS) that include DH-specific descriptors to rate intensity, duration, tolerability and descriptive qualities of the participant’s response [23]. The LMS have been validated for DH and shown to provide advantages over a standard visual analogue scale when assessing DH-associated pain [23].

With clinical efficacy of an anhydrous toothpaste containing 0.454% w/w stannous fluoride (SnF_2_) established in randomized, controlled clinical trials of up to 8 weeks [24, 25], this study was designed to explore impact of long-term twice daily use of this toothpaste on participant-reported OHrQoL outcomes using the DHEQ and other measures in people with DH. A monadic trial design was chosen because of the extended 24-week study duration. Whilst a comparative study design would have enabled between-treatment comparisons, it would have required participants with clinically diagnosed DH to use a fluoride-only control toothpaste, which provides no relief from the pain of dentine hypersensitivity, for almost 6 months. This was considered less ethically appropriate. A positive control study design was also rejected since this would a) have significantly increased the number of participants, given the clinical endpoints selected b) changed the complexity of the statistical analysis plan (e.g., equivalence/non-inferiority) and c) had an alternate null hypothesis. Therefore, while the authors understand that a monadic approach, with no comparator or control, has limitations, this approach was chosen after carefully considering the balance of scientific and ethical requirements and following discussions with external academic advisors.

## Methods

This 24-week, non-comparative clinical study was conducted across two sites at a clinical research facility in Cheshire, UK (Clinicaltrials.gov: NCT02752958, registered on April 27, 2016). The protocol was approved by the South-Central Hampshire B Research Ethics Committee (Reference: 15/SC/0612). The study was conducted in accordance with the Declaration of Helsinki, the International Conference on Harmonization of Technical Requirements for Registration of Pharmaceuticals for Human Use and local laws and regulations. Minor amendments were made to the protocol including addition of a second study site to increase participant recruitment.

### Participants

Participants were aged 18–55 years, in good general health, with a self-reported history of DH between 0.5–10 years. At the screening visit, eligible participants had at least 20 natural teeth and at least two accessible, non-adjacent teeth (incisors, canines or pre-molars) with signs of erosion, abrasion or facial/cervical gingival recession (EAR), a modified gingival index score of 0 adjacent to the test area [26], clinical tooth mobility of ≤1 [27] and a positive response to a qualifying evaporative (air) assessment. At the baseline visit, eligible participants had a minimum of two accessible non-adjacent teeth exhibiting sensitivity, as determined by evaporative (air) assessment (Schiff sensitivity score of ≥2) [5].

Excluding factors included: a chronic debilitating disease that could affect study outcomes; any condition causing dry mouth; tongue/lip piercings; dental implants; treatments that could interfere with pain perception or cause dry mouth or use of antibiotics during the study/within 2 weeks of baseline; pregnancy; breastfeeding; a known/suspected allergy/intolerance to study materials/ingredients; dental prophylaxis or participation in a study or investigational drug use within 4 weeks, desensitising treatment, tooth bleaching or use of a DH-indicated oral care product within 8 weeks, scaling or root planning within 3 months or gross periodontal disease or treatment of such within 12 months of screening.

Specific dentition exclusions for test teeth included those used as partial denture abutments or with full veneers/crowns or orthodontic bands; cracked enamel; sensitive teeth not expected, in the examiner’s opinion, to respond to over-the-counter toothpaste treatment or with contributing aetiologies other than EAR; teeth currently receiving treatment for caries or treated for decay within 12 months of screening or teeth with exposed dentine with deep, defective, or facial restorations.

### Clinical procedures

At the screening visit, participants gave written informed consent and their demographic characteristics, medical history and concomitant medications were recorded. Participants underwent oral soft tissue (OST) and oral hard tissue (OHT) examinations and an evaporative (air) sensitivity test to identify clinically eligible teeth.

Participants who met eligibility criteria were supplied with a regular fluoride toothpaste (1450 ppm fluoride as sodium monofluorophosphate; UK Signal^®^ Family Protection, Unilever plc, Leatherhead, UK) and a toothbrush (Aquafresh^®^ Clean Control [Everyday Clean]; GSK Consumer Healthcare, Brentford, UK) to use twice daily during the acclimatisation period between screening and baseline visits; first use was carried out under supervision. Treatment adherence was assessed through participant-completed diaries. Participants stopped using their regular oral care products throughout the study and could not use any products, including home remedies, for treating sensitive teeth. Use of dental floss is normally excluded from DH clinical studies; however, in this 24-week clinical trial, dental floss was permitted for impacted food removal only. Throughout the study participants could not chew gum and had to delay non-emergency elective dental treatment/prophylaxis. Before the baseline visit and subsequent treatment visits, participants refrained from all oral hygiene procedures and analgesics for ≥8 h, from eating and drinking for ≥4 h, and from excessive alcohol consumption for ≥24 h. Small sips of water were permitted within 4 h (but not 1 h) of each visit.

At the baseline visit, ongoing eligibility was assessed, any adverse events, incidents and medication changes were recorded and acclimatisation toothpaste adherence was evaluated. Participants completed DHEQ Sections 1 and 2 [14] and underwent an LMS training exercise. DHEQ Section 1 Questions (Q)1–6 were to gather information about pre-treatment DH only so results are not presented. Following OST/OHT examinations, sensitivity of all clinically eligible teeth identified was evaluated by an evaporative (air) test. From the teeth that met the qualifying sensitivity assessments, the investigator selected two non-adjacent teeth (‘test teeth’) to be evaluated throughout the study.

To confirm the clinical response to the study toothpaste, and allow benchmarking with previously reported studies, the evaporative (air) sensitivity (Schiff Sensitivity Scale) measure was selected. Yeaple (tactile) measures were not undertaken, maximising participants focus on self-assessment responses. Evaporative (air) sensitivity was assessed by directing a 1 s air blast onto the exposed dentine surface of each test tooth in turn, having first isolated the tooth surface to prevent adjacent teeth or surrounding soft tissue being exposed to the stimulus. The examiner assessed the participant’s observable stimulus response using the 4-point Schiff Sensitivity Scale [5]. In addition, participants also used self-assessment, Label Magnitude Scales (LMS) for DH where they rated the intensity, duration, tolerability and descriptive quality of their response to the evaporative (air) stimulus using 100 mm LMS [23]. Each LMS is anchored at 0 mm with ‘no pain’ and incorporates perceived magnitude of specific descriptors for pain: Intensity (‘dim’, ‘dull’, ‘sharp’, ‘stabbing’); Duration (‘temporary’, ‘quick’, ‘lingering’, ‘chronic’); Tolerability (‘tolerable’, ‘uncomfortable’, ‘unnerving’, ‘unbearable’); Description (‘twinge’, ‘ache’, ‘throbbing’, ‘shooting’).

Eligible participants were supplied with the test toothpaste: 0.454% w/w SnF_2_ (1100 ppm fluoride) (Sensodyne^®^ Repair and Protect Daily Repair Toothpaste, GSK Consumer Healthcare, German marketplace product), with packaging overwrapped in white vinyl to mask identity. Participants applied a full ribbon of toothpaste to the provided toothbrush (replaced at Baseline and Week 12) and brushed for 1 min, twice daily, for 24 weeks, recording each brushing in a provided diary.

First test toothpaste use was carried out under site supervision. At subsequent visits (Weeks 4, 8, 12, 16, 20 and 24), usage adherence was examined based on participant diaries and participants undertook supervised toothbrushing. Participants completed Section 1 (Q7–9 only) and Section 2 of the DHEQ, followed by OST/OHT examinations. The sensitivity of the two test teeth was assessed by response to evaporative (air) stimuli (Schiff Sensitivity Scale and LMS). The sensitivity of any other eligible teeth was also assessed using the Schiff Sensitivity Scale.

### Safety

The safety population included all participants who received at least one study treatment dose. OST abnormalities and adverse events (AEs) were reported from first use of acclimatisation toothpaste until 5 d after last test treatment administration, with intensity graded as mild, moderate or severe. Clinical judgement was exercised to assess any treatment/AE occurrence relationship.

### Statistical analysis

Approximately 75 eligible participants were allocated to the study to ensure approximately 60 completed. This number, based on a previous DHEQ study (unpublished findings), was expected to have at least 90% power to detect significant changes to Week 24 for each DHEQ variable, with a significance level (alpha) of 0.05 using a two-sided one-sample t-test.

Efficacy analyses were performed on a modified intent-to-treat (mITT) population, defined as all randomised participants who received at least one treatment dose and had at least one post-baseline efficacy assessment. The per protocol (PP) population additionally included all participants with no efficacy-affecting protocol deviation. There were no formal success criteria.

Several DHEQ measures were analysed as separate endpoints: Section 1: Q7, Q8, Q9; Section 2: Total score (Q1–34); individual domains assessing Restrictions (Q1–4), Adaptation (Q5–16), Social Impact (Q17–21), Emotional Impact (Q22–29) and Identity (Q30–34); Global Oral Health rating (Q35) and Effect on Life Overall (Q36–39). Each endpoint was analysed using a mixed effect analysis of variance (ANOVA) model, with visit and site as fixed effects and participant as a random effect. Adjusted means were computed for each visit. Post-baseline visits were compared with the baseline visit, with difference of effect calculated along with 95% confidence intervals (CIs) and *p*-values. Visit by site interaction was included as a term and was found significant at the 10% level; therefore, this was included in the model and all model estimates for within and between visits were reported separately for each site and based on results from visit by site interaction. Due to the study’s exploratory nature, no correction for multiple testing was performed.

At each time point, the mean Schiff sensitivity score (two test teeth, all qualifying teeth) and mean LMS scores (test teeth only) as well as change from baseline were calculated. Analysis was performed as above. Assumption of normality and homogeneity of variance in the ANOVA model were investigated and deemed acceptable.

## Results

**Fig. 1:**
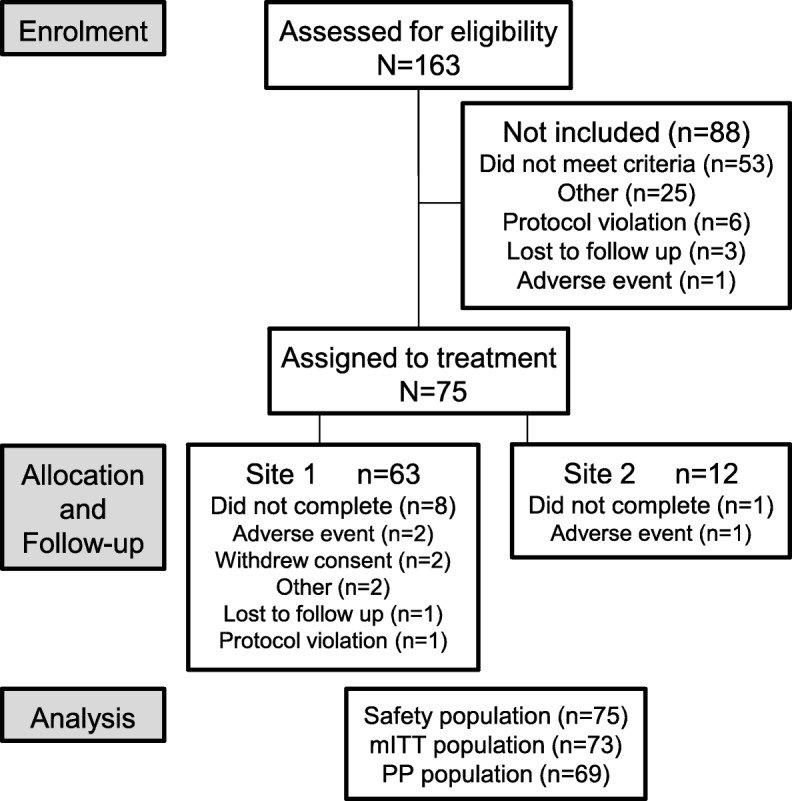
Study flow

The first participant was enrolled on 23 May 2016; the final participant completed the study on 3 February 2017. Of the 163 individuals screened, 75 received at least one dose of test treatment and were included in the safety population (63 at Site 1, 12 at Site 2), 73 were included in the mITT population (Fig. 1). Participants had a mean age of 38.2 (standard deviation: 8.88) and were predominantly female (77.3%). Duration of DH experience was between 6 months and 1 year in six participants (8.2%), 1–5 years in 48 participants (65.8%) and 5–10 years in 19 participants (26.0%).

### Efficacy

#### DHEQ

**Fig. 2:**
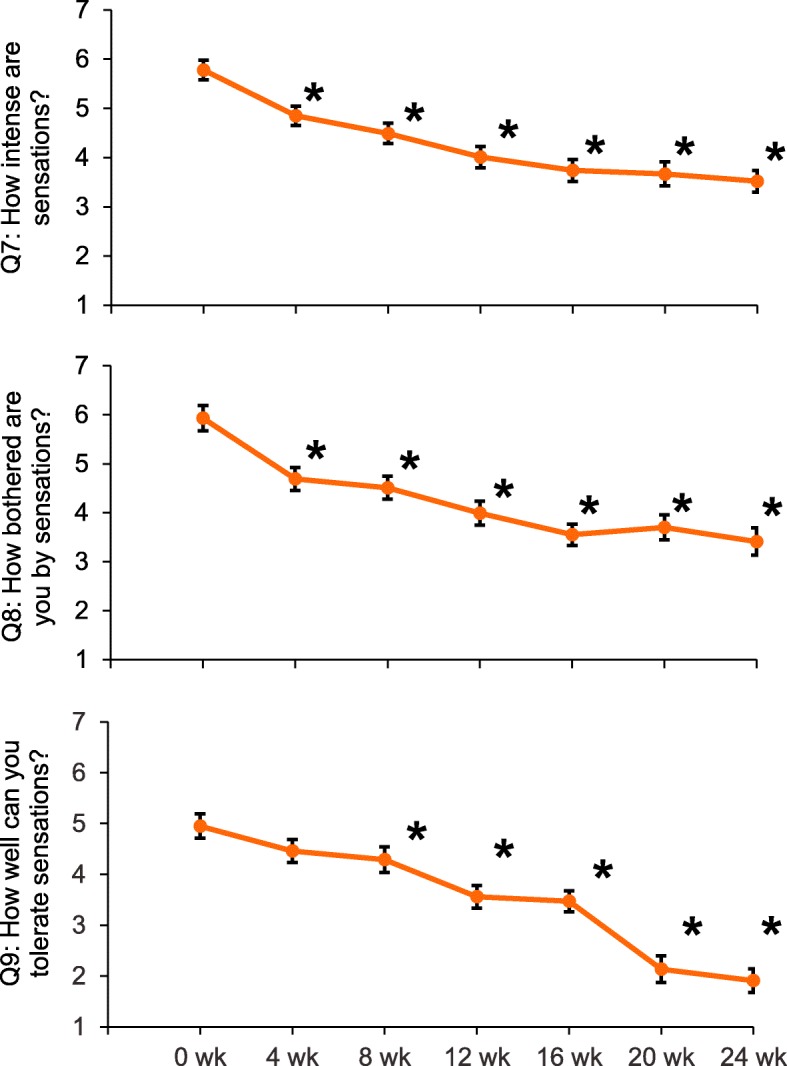
DHEQ Section 1: Raw mean (± SE) scores over time for Q7–9 (mITT population). *p < 0.05 compared to Week 0. Lower scores are favourable; w = week**.** Note: Figures are not to scale: Q7–9 measured on a 1–10 scale

*Section 1:* Ongoing improvements in participant perception of sensitivity were observed over time (Fig. 2). Compared with baseline, these improvements reached statistical significance (*p* < 0.05) at Week 4 for Q7 “How intense were the sensations?” and Q8 “How bothered are you by the sensations?” and at Week 8 for Q9 “How well can you tolerate the sensations?” (Fig. 2; Table 1; Additional file 1: Table S1).

**Table 1:** Adjusted mean change from baseline in DHEQ Section 1, Q7, Q8, Q9 scores at each time point compared to baseline (mITT population)

Week comparison vs baseline	Adjusted mean difference^a^ (95% CI) *p*-value		
	Q7: How intense were the sensations?	Q8: How bothered are you by the sensations?	Q9: How well can you tolerate the sensations?
Week 4	0.88 (0.474, 1.295) **< 0.0001**	1.18 (0.703, 1.660) **< 0.0001**	0.43 (− 0.050, 0.919) 0.0786
Week 8	1.28 (0.875, 1.690) **< 0.0001**	1.45 (0.977, 1.926) **< 0.0001**	0.69 (0.210, 1.717) **0.0050**
Week 12	1.74 (1.333, 2.145) **< 0.0001**	1.95 (1.473, 2.418) **< 0.0001**	1.40 (0.918, 1.875) **< 0.0001**
Week 16	1.96 (1.545, 2.371) **< 0.0001**	2.33 (1.848, 2.810) **< 0.0001**	1.44 (0.951, 1.926) **< 0.0001**
Week 20	2.06 (1.643, 2.469) **< 0.0001**	2.21 (1.732, 2.694) **< 0.0001**	1.17 (0.678, 1.653) **< 0.0001**
Week 24	2.21 (1.794, 2.620) **< 0.0001**	2.50 (2.020, 2.982) **< 0.0001**	1.56 (1.072, 2.047) **< 0.0001**

^a^Difference is the baseline score minus respective Week score such that a positive difference shows an improvement in score; *p*-values in bold are significant

**Fig. 3:**
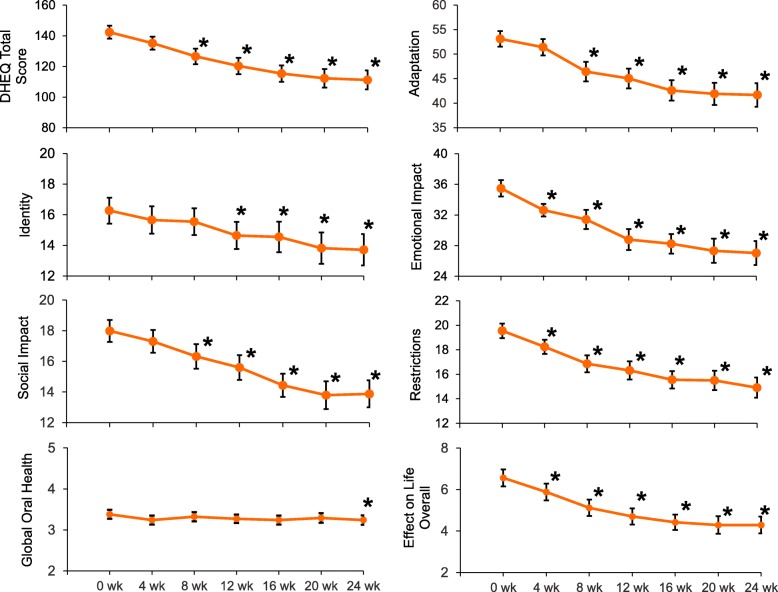
DHEQ Section 2: (mITT population). Raw mean (± standard error) scores over time for DHEQ Total Score (Q1–34), individual DHEQ domain scores, Global Oral Health Rating (Q35) and Effect on Life Overall (Q36–39). *p < 0.05 compared to Week 0. Lower scores are favourable; w = week. Note: Figures are not to scale: Total DHEQ score = 34–238 point scale; Adaptation = 12–84 point scale; Identity and Social Impact = 5–35 point scale; Emotional Impact = 8–56 point scale; Restrictions = 4–28 point scale; Global Oral Health = 1–6 scale; Effect on Life Overall = 0–16 scale

*Section 2:* Ongoing improvements in participant perception of the impact of DH (DHEQ Total Score) were observed over time (Fig. 3), reaching statistical significance compared with baseline from Week 8 onwards (all *p* < 0.0001 vs baseline; Table 2).

**Table 2:** Adjusted mean difference in change from baseline in mean total DHEQ score, Schiff score and LMS scores at each time point compared to baseline (mITT population)

Week comparison vs baseline	Adjusted mean difference^a^ (95% CI) *p*-value			
	Total DHEQ score	Schiff score (test teeth)	Schiff score (all qualifying)	
Week 4	5.89 (−1.018, 12.806) 0.0944	1.09 (0.940, 1.250) **< 0.0001**	0.62 (0.533, 0.715) **< 0.0001**	
Week 8	16.99 (10.128, 23.849) **< 0.0001**	1.23 (1.077, 1.383) **< 0.0001**	0.71 (0.620, 0.800) **< 0.0001**	
Week 12	22.70 (15.870, 29.539) **< 0.0001**	1.35 (1.198, 1.501) **< 0.0001**	0.84 (0.753, 0.931) **< 0.0001**	
Week 16	27.21 (20.245, 34.165) **< 0.0001**	1.40 (1.248, 1.559) **< 0.0001**	0.90 (0.804, 0.986) **< 0.0001**	
Week 20	31.42 (24.464, 38.385) **< 0.0001**	1.50 (1.349, 1.658) **< 0.0001**	0.96 (0.864, 1.046) **< 0.0001**	
Week 24	32.55 (25.585, 39.506) **< 0.0001**	1.66 (1.503, 1.812) **< 0.0001**	1.09 (0.995, 1.777) **< 0.0001**	
	**LMS Intensity**	**LMS Duration**	**LMS Tolerability**	**LMS Description**
Week 4	13.27 (9.226, 17.306) **< 0.0001**	12.94 (9.111, 16.762) **< 0.0001**	15.57 (12.171, 18.973) **< 0.0001**	18.35 (13.850, 22.855) **< 0.0001**
Week 8	17.72 (13.733, 21.704) **< 0.0001**	15.83 (12.054, 19.603) **< 0.0001**	16.63 (13.273, 19.983) **< 0.0001**	19.98 (15.536, 24.421) **< 0.0001**
Week 12	20.87 (16.920, 24.829) **< 0.0001**	19.07 (15.326, 22.816) **< 0.0001**	20.29 (16.961, 23.619) **< 0.0001**	24.32 (19.916, 28.730) **< 0.0001**
Week 16	26.10 (22.051, 30.144) **< 0.0001**	22.92 (19.090, 26.754) **< 0.0001**	24.06 (20.652, 27.465) **< 0.0001**	26.16 (21.654, 30.674) **< 0.0001**
Week 20	27.74 (23.716, 31.771) **< 0.0001**	25.51 (21.695, 29.323) **< 0.0001**	25.55 (22.155, 28.936) **< 0.0001**	28.28 (23.793, 32.771) **< 0.0001**
Week 24	29.86 (25.833, 33.888) **< 0.0001**	28.74 (24.930, 32.558) **< 0.0001**	26.75 (23.359, 30.140) **< 0.0001**	30.83 (26.341, 35.319) **< 0.0001**

^a^Difference is the baseline score minus Week score such that a positive difference shows an improvement in score; *p*-values in bold are significant

Ongoing improvements in the Section 2 domains were also observed (Fig. 3). Compared with baseline, improvements reached statistical significance (*p* < 0.05) at Week 4 for Restrictions and Emotional Impact, Week 8 for Adaptation and Social Impact and Week 12 for Identity (Fig. 3; Table 3).

**Table 3:** Adjusted mean change from baseline in DHEQ Section 2 scores: Total score (Q1–34), Restrictions (Q1–4), Adaptation (Q5–16), Social Impact (Q17–21), Emotional Impact (Q22–29), Identity (Q30–34), Global Oral Health rating (Q35), Effect on Life Overall (Q36–39) at each time point compared to baseline (mITT population)

Week comparison vs baseline	Adjusted mean difference^a^ (95% CI) *p*-value			
	Restrictions	Adaptation	Social Impact	Emotional Impact
Week 4	1.18 (0.108, 2.262) **0.0312**	1.11 (−1.592, 3.821) 0.4185	0.57 (−0.604, 1.738) 0.3415	2.42 (0.483, 4.352) **0.0145**
Week 8	2.08 (1.728, 3.866) **< 0.0001**	7.08 (4.391, 9.764) **< 0.0001**	1.77 (0.610, 2.935) **0.0029**	4.42 (2.498, 6.339) **< 0.0001**
Week 12	3.26 (2.192, 4.322) **< 0.0001**	8.35 (5.674, 11.026) **< 0.0001**	2.42 (1.266, 3.582) **< 0.0001**	6.85 (4.933, 8.759) **< 0.0001**
Week 16	3.93 (2.844, 5.012) **< 0.0001**	10.43 (7.708, 13.158) **< 0.0001**	3.55 (2.371, 4.729) **< 0.0001**	7.17 (5.219, 9.115) **< 0.0001**
Week 20	4.15 (3.063, 5.232) **< 0.0001**	11.73 (9.008, 14.459) **< 0.0001**	4.39 (3.210, 5.568) **< 0.0001**	8.39 (6.438, 10.335) **< 0.0001**
Week 24	4.74 (3.654, 5.823) **< 0.0001**	11.96 (9.235, 14.686) **< 0.0001**	4.30 (3.119, 5.477) **< 0.0001**	8.67 (6.726, 10.623) **< 0.0001**
	**Identity**	**Global Oral Health Rating**	**Effect on Life Overall**	
Week 4	0.63 (−0.738, 1.989) 0.3676	0.09 (−0.059, 0.243) 0.2326	0.60 (0.002, 1.194) **0.0493**	
Week 8	0.90 (−0.451, 2.255) 0.1907	0.07 (−0.085, 0.216) 0.3934	1.42 (0.830, 2.013) **< 0.0001**	
Week 12	1.81 (0.467, 3.162) **0.0085**	0.13 (−0.016, 0.284) 0.0785	1.87 (1.278, 2.457) **< 0.0001**	
Week 16	2.12 (0.744, 3.489) **0.0026**	0.14 (−0.011, 0.294) 0.0682	2.03 1.429, 2.629) **< 0.0001**	
Week 20	2.74 (1.371, 4.116) **0.0001**	0.13 (−0.018, 0.287) 0.0844	2.34 (1.743, 2.943) **< 0.0001**	
Week 24	2.85 (1.477, 4.222) **< 0.0001**	0.18 (0.027, 0.332) **0.0211**	2.34 (1.743, 2.943) **< 0.0001**	

^a^Difference is the baseline score minus Week score such that a positive difference shows an improvement in score; *p*-values in bold are significant

Mean Global Oral Health score showed a statistically significant difference at Week 24 only while Mean Effect on Life Overall scores showed an improvement over time, reaching statistical significance compared with baseline at Week 4 (*p* < 0.05) (Fig. 3; Table 3).

### Schiff sensitivity scale and LMS scores

**Fig. 4:**
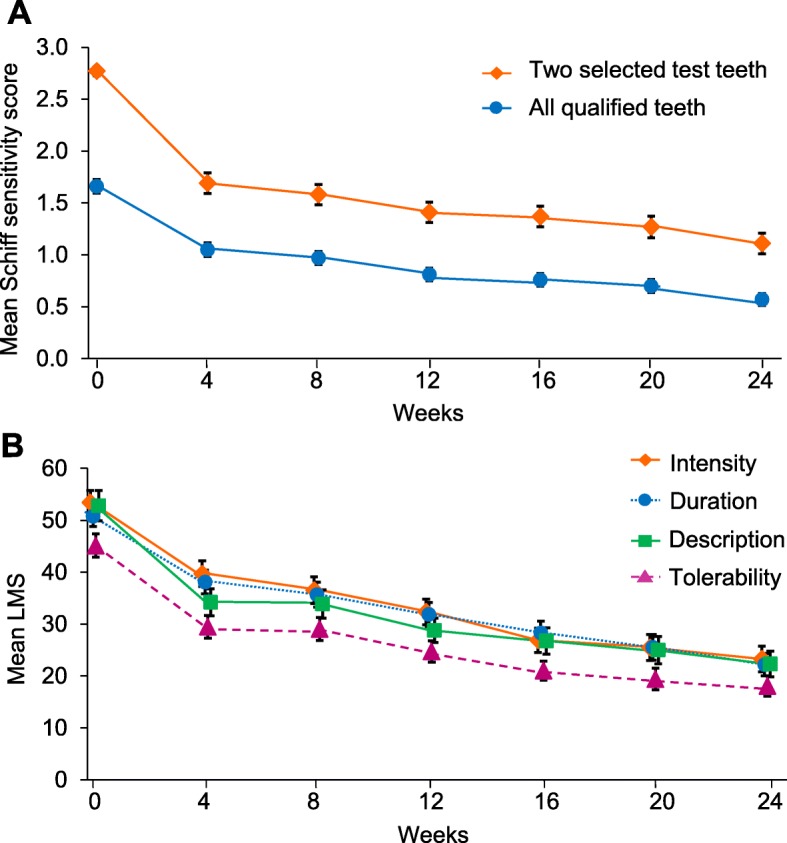
Raw mean (±SE) scores over time (mITT population). **a** evaporative (air) sensitivity (Schiff Sensitivity Scale score) for the two test teeth and all qualifying teeth and **b** Labelled Magnitude Scales for the two test teeth

Mean Schiff sensitivity scores improved over time for the two test teeth and for all qualifying teeth at screening, reaching statistical significance compared with baseline from Week 4 onwards (*p* < 0.0001 for all) (Fig. 4a; Table 1). Mean LMS scores for each of the four scales (Intensity, Duration, Tolerability, Description) improved over time for the two test teeth, reaching statistical significance compared with baseline from Week 4 onwards (*p* < 0.0001 for all) (Fig. 4b; Table 1).

### Analysis of efficacy endpoints by site

There was a consistently greater improvement in efficacy variables in participants enrolled at Site 1 (*n* = 61) compared with Site 2 (*n* = 12), except for Global Oral Health and Effect on Life Overall ratings of the Site 2 scores, which showed no clear overall pattern (data not shown).

### Safety

There were 105 treatment-emergent AEs (TEAEs) reported by 47 participants (62.7%). Of these TEAEs, 10 were considered treatment-related: Oral mucosal exfoliation (*n* = 4); oral mucosal erythema (*n* = 2); aphthous ulcer (*n* = 1); oral discomfort (*n* = 1); lip exfoliation (*n* = 1); mouth ulcerations (*n* = 1). All but three (two of moderate oral mucosal desquamation, one of lip exfoliation) were mild in intensity. Two participants withdrew because of TEAEs: one with oral mucosal exfoliation and oral mucosal erythema, one with oral mucosal exfoliation. All treatment-related TEAEs resolved by study end. No TEAEs were serious and no medical device incidents were reported.

## Discussion

This long-term study in individuals with DH investigated the impact on OHrQoL of twice daily brushing with an anhydrous SnF_2_-based toothpaste. While clinical efficacy (up to 8 weeks) has previously been demonstrated for this toothpaste in randomized controlled clinical trials [24, 25, 28], this is the first study evaluating its longer-term benefits (24 weeks). This study had a relatively large sample size and number of participants who completed the study, adding validity to the results.

Overall, the psychosocial OHrQoL results paralleled the biomedical results observed in this and other clinical trials [24, 25, 28]. Pain assessment results confirmed the performance of the DH-targeted toothpaste, in line with literature reported RCT’s, with change from baseline of DH statistically significant after 4 weeks and a continued decline in Schiff sensitivity scores throughout the study. In comparison to the previous studies of this toothpaste [24, 25], at 8 weeks use, changes from baseline were of a similar magnitude, dropping below the score of ‘2’ needed to rate a tooth as being hypersensitive. The baseline participant-reported LMS data was similar to that shown in a dental practice-based study [29] and results here showed that all the LMS themes questioned regarding pain (Description, Duration, Intensity, Tolerability) decreased significantly over the 24 weeks.

While pain assessments are standard for a clinical trial to show treatment efficacy, DH can also be described as a set of sensations including ‘itching’ and ‘shivering’ and like ‘needles’ or ‘brain freeze’ [7]. Impact of these sensations on a study participant’s everyday life was specifically explored with the DHEQ. Responses to DHEQ Section 1 questions, which examine physical impact of DH, showed statistically significant improvements from 4 or 8 weeks treatment indicating that over the course of the study, sensations were rated as less intense, less bothersome and more tolerable.

Awareness that DH might occur can increase a person’s pain-avoiding habits [7]. As such, decreases in scores assessing DH impact are favourable when examining a treatment’s effectiveness. This was reflected in decreases to scores in DHEQ Section 2, which examines ways in which DH affects a person’s daily life. Statistically significant improvement in OHrQoL was demonstrated through reductions in mean DHEQ Total Scores after 8 weeks, which continued to decrease with time. The observation that this score still declined between the final two assessment visits suggests that further improvements in DH relief may be possible with use of this toothpaste for longer than 24 weeks. The ‘minimally important difference’ (the smallest difference in a score that a person perceives as important for the DHEQ Total Score) has been estimated as being between 22 and 29 points from analysis of three separate studies [13]. Here, the change in mean DHEQ Total Score from baseline was greater than 22 points from Week 12 onward, demonstrating that the improvement in OHrQoL observed was likely to be meaningful to the participants. The Effect on Life Overall subscale also improved over time, with statistically significant improvements after 4 weeks.

Improvements were shown in all DHEQ Section 2 OHrQoL domains. Pain and physical impact decrease was reflected from 4 weeks’ treatment in the Restrictions domain, which questioned issues participants encountered related to eating. It has been shown previously that modifying eating and drinking habits may be a negative consequence of DH [13]. This study confirms that this need can be reduced by twice daily brushing with the anti-sensitivity toothpaste used here. The Adaptations domain showed a statistically significant improvement after 8 weeks. As this domain informs on how individuals avoid stimuli that provoke DH (foods in particular) and on coping strategies employed to mitigate effects of these stimuli, improvement in this domain is expected to follow improvements in the Restrictions domain. Likewise, the Social Impact domain informs on restrictions participants impose on themselves when eating/interacting with others and how this impacts them in a social setting; statistically significant improvements were demonstrated in this domain after 8 weeks.

The Emotional Impact domain, which pays regard to anxiety and annoyance that individuals perceive from their DH, showed statistically significant improvements from baseline after 4 weeks. Emotional impact has previously been reported to be a component of DH [7]; hence, it is important that treatment with an anti-sensitivity toothpaste was shown to decrease this domain score.

Twelve weeks was required before a statistically significant improvement in the Identity domain was demonstrated, consistent with previous studies where Identity was generally the domain with the least change from baseline [13]. As this domain relates to how an individual perceives themselves in the context of their health and/or age, it is possible that this self-perception domain is slower to change than more tangible areas such as eating restrictions/adaptations. Interestingly, the Global Oral Health question showed little improvement until Week 24. This question has previously been shown to correlate poorly with clinically derived sensitivity assessments such as the Schiff Sensitivity Score [30]. Further, the same study demonstrated little longitudinal change in responses to this question, mirroring this study. This suggests that participants perhaps did not perceive a strong relationship between their DH symptoms and their overall oral health.

An explanation for the by-site differences observed is not obvious. The sites used the same clinical examiner and are geographically close. Participant demographics were similar, though Site 2 enrolled slightly older participants who had greater previous DH study experience. There were minor differences in baseline characteristics; however, it not clear how these could influence toothpaste efficacy. It is more likely that differences were due to random effects owing to the small sample size at Site 2 (*n* = 12) compared to Site 1 (*n* = 63).

## Conclusions

In conclusion, long-term twice daily use of a 0.454% w/w SnF_2_ anti-sensitivity toothpaste provides an important range of clinically proven oral health benefits together with a beneficial and increasing positive impact on OHrQoL measures. The study treatment was generally well tolerated.

## Supplementary information

**Additional file 1: Table S1.** Adjusted mean change from baseline in DHEQ Section 1, Q7, Q8, Q9 scores at each time point compared to baseline (ITT population). **Table S2.** Adjusted mean change from baseline in DHEQ Section 2 scores: Total score (Q1–34), Restrictions (Q1–4), Adaptation (Q5–16), Social Impact (Q17–21), Emotional Impact (Q22–29), Identity (Q30–34), Global Oral Health rating (Q35), Effect on Life Overall (Q36–39) at each time point compared to baseline (ITT population)

## Data Availability

Within 6 months of this publication, anonymised individual participant data, the annotated case report form, protocol, reporting and analysis plan, data set specifications, raw dataset, analysis-ready dataset, and clinical study report will be available for research proposals approved by an independent review committee. Proposals should be submitted to www.clinicalstudydatarequest.com. A data access agreement will be required.

## Abbreviations

AE: Adverse event
ANOVA: Analysis of variance
CIs: Confidence intervals
DH: Dentine hypersensitivity
DHEQ: Dentin Hypersensitivity Experience Questionnaire
EAR: Erosion, abrasion or facial/cervical gingival recession
LMS: Labelled Magnitude Scales
mITT: modified intent-to-treat
OHIP: Oral Health Impact Profile
OHrQoL: Oral health-related quality of life
OHT: Oral hard tissue
OST: Oral soft tissue
PP: Per protocol
Q: Question
SE: Standard error
SnF_2_: Stannous fluoride
TEAEs: Treatment-emergent adverse events
