# Supplementary material for: Impact of toothpaste on oral health-related quality of life in people with dentine hypersensitivity
Source: BMC Oral Health. 2019 Oct 22;19:226. doi: 10.1186/s12903-019-0919-x (PMC6805594; doi:10.1186/s12903-019-0919-x)
Supplement: Supplementary file 1 — Additional file 1: Table S1. Adjusted mean change from baseline in DHEQ Section 1, Q7, Q8, Q9 scores at each time point compared to baseline (ITT population). Table S2. Adjusted mean change from baseline in DHEQ Section 2 scores: Total score (Q1–34), Restrictions (Q1–4), Adaptation (Q5–16), Social Impact (Q17–21), Emotional Impact (Q22–29), Identity (Q30–34), Global Oral Health rating (Q35), Effect on Life Overall (Q36–39) at each time point compared to baseline (ITT population) [file 12903_2019_919_MOESM1_ESM.docx]

**Impact of toothpaste on oral health-related quality of life in people with dentine hypersensitivity**

Stephen Mason, Gary R. Burnett, Nisha Patel, Avinash Patil, Robert Maclure

**Table S1.** Adjusted mean change from baseline in DHEQ Section 1, Q7, Q8, Q9 scores at each time point compared to baseline (ITT population)

| **Week comparison vs baseline** | **Adjusted mean difference^a^ (95% CI) p-value** | | |
| --- | --- | --- | --- |
|  | **Q7: How intense were the sensations?** | **Q8: How bothered are you by the sensations?** | **Q9: How well can you tolerate the sensations?** |
| **Week 4** | 0.88 (0.474, 1.295) <0.0001 | 1.18 (0.703, 1.660) <0.0001 | 0.43 (-0.050, 0.919) 0.0786 |
| **Week 8** | 1.28 (0.875, 1.690) <0.0001 | 1.45 (0.977, 1.926) <0.0001 | 0.69 (0.210, 1.717) 0.0050 |
| **Week 12** | 1.74 (1.333, 2.145) <0.0001 | 1.95 (1.473, 2.418) <0.0001 | 1.40 (0.918, 1.875) <0.0001 |
| **Week 16** | 1.96 (1.545, 2.371) <0.0001 | 2.33 (1.848, 2.810) <0.0001 | 1.44 (0.951, 1.926) <0.0001 |
| **Week 20** | 2.06 (1.643, 2.469) <0.0001 | 2.21 (1.732, 2.694) <0.0001 | 1.17 (0.678, 1.653) <0.0001 |
| **Week 24** | 2.21 (1.794, 2.620) <0.0001 | 2.50 (2.020, 2.982) <0.0001 | 1.56 (1.072, 2.047) <0.0001 |

a. Difference is the baseline score minus Week score such that a positive difference shows an improvement in score

**Table S2.** Adjusted mean change from baseline in DHEQ Section 2 scores: Total score (Q1–34), Restrictions (Q1–4), Adaptation (Q5–16), Social Impact (Q17–21), Emotional Impact (Q22–29), Identity (Q30–34), Global Oral Health rating (Q35), Effect on Life Overall (Q36–39) at each time point compared to baseline (ITT population)

| **Week comparison vs baseline** | **Adjusted mean difference^a^ (95% CI) p-value** | | | | |
| --- | --- | --- | --- | --- | --- |
|  | **Restrictions** | **Adaptation** | **Social Impact** | **Emotional Impact** |  |
| **Week 4** | 1.18 (0.108, 2.262) 0.0312 | 1.11 (-1.592, 3.821) 0.4185 | 0.57 (-0.604, 1.738) 0.3415 | 2.42 (0.483, 4.352) 0.0145 |  |
| **Week 8** | 2.08 (1.728, 3.866) <0.0001 | 7.08 (4.391, 9.764) <0.0001 | 1.77 (0.610, 2.935) 0.0029 | 4.42 (2.498, 6.339) <0.0001 |  |
| **Week 12** | 3.26 (2.192, 4.322) <0.0001 | 8.35 (5.674, 11.026) <0.0001 | 2.42 (1.266, 3.582) <0.0001 | 6.85 (4.933, 8.759) <0.0001 |  |
| **Week 16** | 3.93 (2.844, 5.012) <0.0001 | 10.43 (7.708, 13.158) <0.0001 | 3.55 (2.371, 4.729) <0.0001 | 7.17 (5.219, 9.115) <0.0001 |  |
| **Week 20** | 4.15 (3.063, 5.232) <0.0001 | 11.73 (9.008, 14.459) <0.0001 | 4.39 (3.210, 5.568) <0.0001 | 8.39 (6.438, 10.335) <0.0001 |  |
| **Week 24** | 4.74 (3.654, 5.823) <0.0001 | 11.96 (9.235, 14.686) <0.0001 | 4.30 (3.119, 5.477) <0.0001 | 8.67 (6.726, 10.623) <0.0001 |  |
|  | **Identity** | **Global Oral Health Rating** | **Effect on Life Overall** |  |  |
| **Week 4** | 0.63 (-0.738, 1.989) 0.3676 | 0.09 (-0.059, 0.243) 0.2326 | 0.60 (0.002, 1.194) 0.0493 |  |  |
| **Week 8** | 0.90 (-0.451, 2.255) 0.1907 | 0.07 (-0.085, 0.216) 0.3934 | 1.42 (0.830, 2.013) <0.0001 |  |  |
| **Week 12** | 1.81 (0.467, 3.162) 0.0085 | 0.13 (-0.016, 0.284) 0.0785 | 1.87 (1.278, 2.457) <0.0001 |  |  |
| **Week 16** | 2.12 (0.744, 3.489) 0.0026 | 0.14 (-0.011, 0.294) 0.0682 | 2.03 1.429, 2.629) <0.0001 |  |  |
| **Week 20** | 2.74 (1.371, 4.116) 0.0001 | 0.13 (-0.018, 0.287) 0.0844 | 2.34 (1.743, 2.943) <0.0001 |  |  |
| **Week 24** | 2.85 (1.477, 4.222) <0.0001 | 0.18 (0.027, 0.332) 0.0211 | 2.34 (1.743, 2.943) <0.0001 |  |  |

a. Difference is the baseline score minus Week score such that a positive difference shows an improvement in score
